# Supplementary material for: Modeling glioblastoma heterogeneity as a dynamic network of cell states
Source: Mol Syst Biol. 2021 Sep 16;17(9):e10105. doi: 10.15252/msb.202010105 (PMC8444284; doi:10.15252/msb.202010105)
Supplement: Supplementary file 5 — Source Data for Figure 3 [file MSB-17-e10105-s001.zip › Figure3A_sourcedata/GSEA_3065/nosigdb_state4.GseaPreranked.1622718430994/TISIG_PMID_32649888.html]

Details for gene set TISIG\_PMID\_32649888[GSEA]

|  || Dataset | state4 |
| Phenotype | NoPhenotypeAvailable |
| Upregulated in class | na\_pos |
| GeneSet | TISIG\_PMID\_32649888 |
| Enrichment Score (ES) | 0.6325736 |
| Normalized Enrichment Score (NES) | 2.1590645 |
| Nominal p-value | 0.0 |
| FDR q-value | 1.8888888E-4 |
| FWER p-Value | 0.001 |
Table: GSEA Results Summary

  

Fig 1: Enrichment plot: TISIG\_PMID\_32649888      
 Profile of the Running ES Score & Positions of GeneSet Members on the Rank Ordered List

  

| PROBE | GENE SYMBOL | GENE\_TITLE | RANK IN GENE LIST | RANK METRIC SCORE | RUNNING ES | CORE ENRICHMENT || 1 | FABP7 |  |  | 5 | 0.656 | 0.1664 | Yes |
| 2 | HOPX |  |  | 28 | 0.360 | 0.2546 | Yes |
| 3 | SOX9 |  |  | 48 | 0.299 | 0.3276 | Yes |
| 4 | TTYH1 |  |  | 100 | 0.249 | 0.3829 | Yes |
| 5 | ATP1B2 |  |  | 159 | 0.217 | 0.4289 | Yes |
| 6 | EDNRB |  |  | 271 | 0.180 | 0.4571 | Yes |
| 7 | NDRG4 |  |  | 384 | 0.157 | 0.4792 | Yes |
| 8 | RASSF2 |  |  | 564 | 0.129 | 0.4834 | Yes |
| 9 | MLC1 |  |  | 594 | 0.126 | 0.5109 | Yes |
| 10 | KCNIP1 |  |  | 603 | 0.125 | 0.5415 | Yes |
| 11 | WSCD1 |  |  | 605 | 0.125 | 0.5731 | Yes |
| 12 | C1orf61 |  |  | 803 | 0.104 | 0.5679 | Yes |
| 13 | BBOX1 |  |  | 823 | 0.102 | 0.5908 | Yes |
| 14 | ZIC1 |  |  | 1018 | 0.087 | 0.5818 | Yes |
| 15 | CSPG5 |  |  | 1035 | 0.086 | 0.6011 | Yes |
| 16 | SLC1A3 |  |  | 1069 | 0.083 | 0.6170 | Yes |
| 17 | AGT |  |  | 1103 | 0.082 | 0.6326 | Yes |
| 18 | TRIB2 |  |  | 1496 | 0.062 | 0.5855 | No |
| 19 | LRP4 |  |  | 1502 | 0.062 | 0.6004 | No |
| 20 | POU3F2 |  |  | 1531 | 0.061 | 0.6114 | No |
| 21 | NES |  |  | 1551 | 0.060 | 0.6236 | No |
| 22 | CKB |  |  | 1774 | 0.053 | 0.6015 | No |
| 23 | SDC3 |  |  | 2214 | 0.041 | 0.5416 | No |
| 24 | KCNJ16 |  |  | 2305 | 0.039 | 0.5371 | No |
| 25 | CCND2 |  |  | 2367 | 0.038 | 0.5369 | No |
| 26 | FGFR3 |  |  | 2779 | 0.029 | 0.4783 | No |
| 27 | GAS1 |  |  | 3553 | 0.011 | 0.3570 | No |
| 28 | CDH4 |  |  | 3676 | 0.006 | 0.3390 | No |
| 29 | ARC |  |  | 4942 | -0.078 | 0.1560 | No |
| 30 | DPP6 |  |  | 5279 | -0.108 | 0.1296 | No |
| 31 | EGFR |  |  | 5316 | -0.110 | 0.1519 | No |
Table: GSEA details [plain text format]

  

Fig 2: TISIG\_PMID\_32649888: Random ES distribution      
 Gene set null distribution of ES for **TISIG\_PMID\_32649888**
